# Conservative Management of a Non-vital, Young, Permanent Tooth in a Pediatric Patient: A Case Report

**DOI:** 10.7759/cureus.64709

**Published:** 2024-07-17

**Authors:** Namrata O Mangtani, Sakshi P Kabra, Nilima R Thosar, Meenal S Pande, Neha Pankey, Dhruvi Solanki

**Affiliations:** 1 Dentistry, Sharad Pawar Dental College and Hospital, Datta Meghe Institute of Higher Education and Research (Deemed to be University), Wardha, IND; 2 Pediatric and Preventive Dentistry, Sharad Pawar Dental College and Hospital, Datta Meghe Institute of Higher Education and Research (Deemed to be University), Wardha, IND; 3 Pediatric and Preventive Dentistry, Sharad Pawar Dental College and Hospital, Datta Meghe Institute of Higher Education and Research (Deemed to be University), wardha, IND

**Keywords:** dental fracture, ellis class iv fracture, pulp vitality test, pulp therapy, non-vital rct

## Abstract

A fracture due to trauma in children is one of the most common traumas treated by dentists. Pain, swelling, and aesthetics are some of the most common chief complaints patients report to dental clinics after trauma. The anterior teeth play a significant role in the aesthetics of the patient's smile thus affecting the patient's psychological well-being. In addition, they are also important for mastication and speech. Treatment of fractured teeth, especially in the anterior region, has always been a major concern of aesthetics for the patient and the dentist. The most commonly observed fracture, i.e., Ellis class IV fracture includes endodontic treatment due to exposure of the pulp during trauma. This brief case report presents a case of an 11-year-old female with the chief complaint of a fractured tooth in the maxillary front teeth region. The treatment plan included the preventive procedures followed by root canal treatment and composite restoration with the right permanent maxillary lateral incisor. The benefit of this is directly linked to the professional's dexterity, skill, and technical mastery, as well as the achievement of desirable outcomes.

## Introduction

Children most commonly experience trauma to oral hard and soft tissues. Traumatic dental injuries are most commonly observed among all facial injuries, showing a prevalence of 22% in primary dentition and 15% in permanent dentition, along with an incidence rate of 28.2 cases per 1,000 per year [1]. The high prevalence rate of traumatic injuries observed in children is due to poor sensory-motor coordination and poor skills in measuring risks, making them the most prevalent age group for traumatic injuries. Traumatic dental injuries in children around the world are usually caused by accidental falls, injuries during sports activities, and road traffic accidents. These injuries are most commonly observed in the maxillary anterior region of the jaw [2]. This has a significant impact on the psychological and physical health of the child. The younger population is more concerned about missing/fractured/discolored anterior teeth and is often very conscious about their smile aesthetics. The anterior teeth play a significant role in the aesthetics of the patient's smile, thus affecting the patient's psychological well-being. In addition, they are also important for mastication and speech [3].

## Case presentation

**Figure 1:**
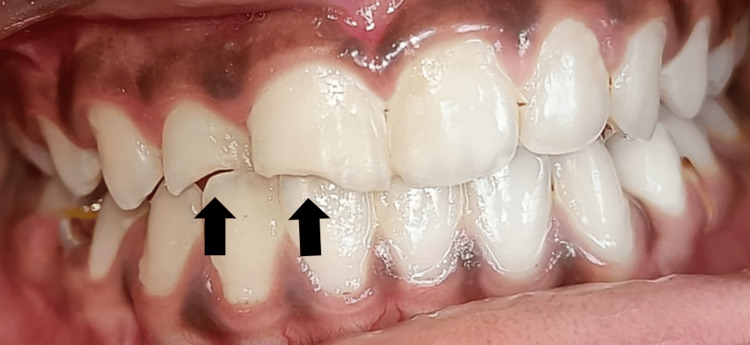
Pre-operative image showing an Ellis class IV fracture with a right permanent maxillary lateral incisor, an Ellis class I fracture with a right permanent maxillary central incisor, grossly decayed right deciduous mandibular first molar, and over-retained right deciduous maxillary first molar

An 11-year-old female child visited the Department of Pediatric Dentistry with the chief complaint of pain in the upper right anterior region of the jaw for 15 days. The patient was apparently alright 15 days ago when she fell from a swing and fractured her tooth. There was a history of pain in the right maxillary lateral incisor, which was of moderate intensity, intermittent in nature, and increased on mastication and consumption of hot or cold drinks. She gave no history of swelling, nausea, vomiting, or bleeding after the fall. The clinical investigation showed a fracture involving the enamel, dentin, and pulp with the right permanent maxillary lateral incisor. This was associated with grade I mobility and tenderness on percussion was negative. On electric pulp testing (EPT), no response was seen with respect to the right permanent maxillary lateral incisor, indicating a non-vital tooth. Clinical examination of extraoral features showed bilateral facial symmetry and normal-appearing facial skin. Lymph nodes were non-palpable, lips were competent, and temporomandibular joint (TMJ) movement was bilaterally synchronous and non-tender. Hard tissue examination showed an Ellis class IV fracture with a right maxillary lateral incisor and Grade I mobility with the right maxillary lateral incisor. An Ellis class I fracture with a right permanent maxillary central incisor, a grossly decayed right deciduous mandibular first molar, and an over-retained right deciduous maxillary first molar were seen. No soft tissue injury was observed (Figure 1).

For radiographic investigation, an intraoral periapical radiograph (IOPA) was advised to the child, which revealed a discontinuous lamina dura associated with the right permanent maxillary lateral incisor. Hence, based on the clinical and radiographic evaluation, the final diagnosis of this case was an Ellis class IV fracture with a right permanent maxillary lateral incisor and an Ellis class I fracture with a right permanent maxillary central incisor.

**Figure 2:**
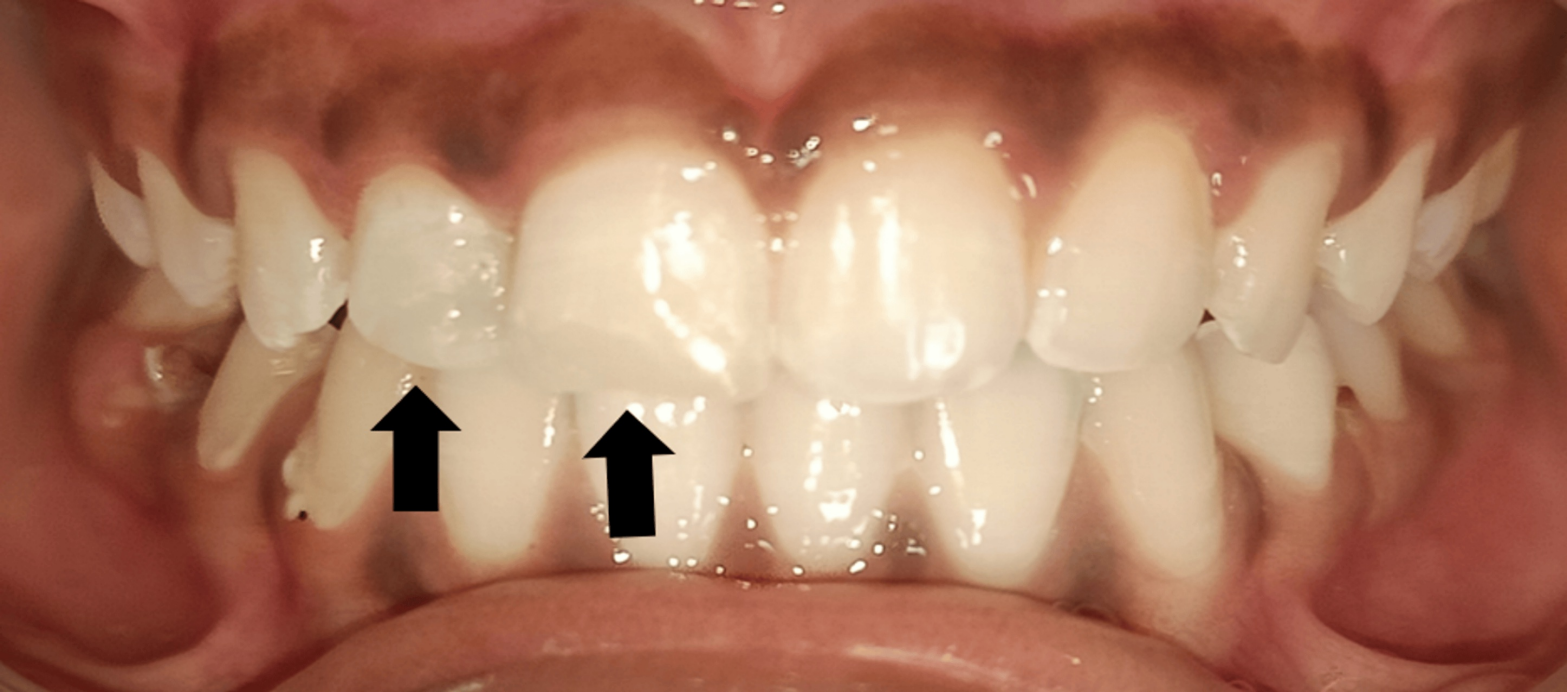
Postoperative image showing root canal treatment followed by composite restoration with the right permanent maxillary lateral incisor and composite restoration with the right permanent maxillary central incisor

**Figure 3:**
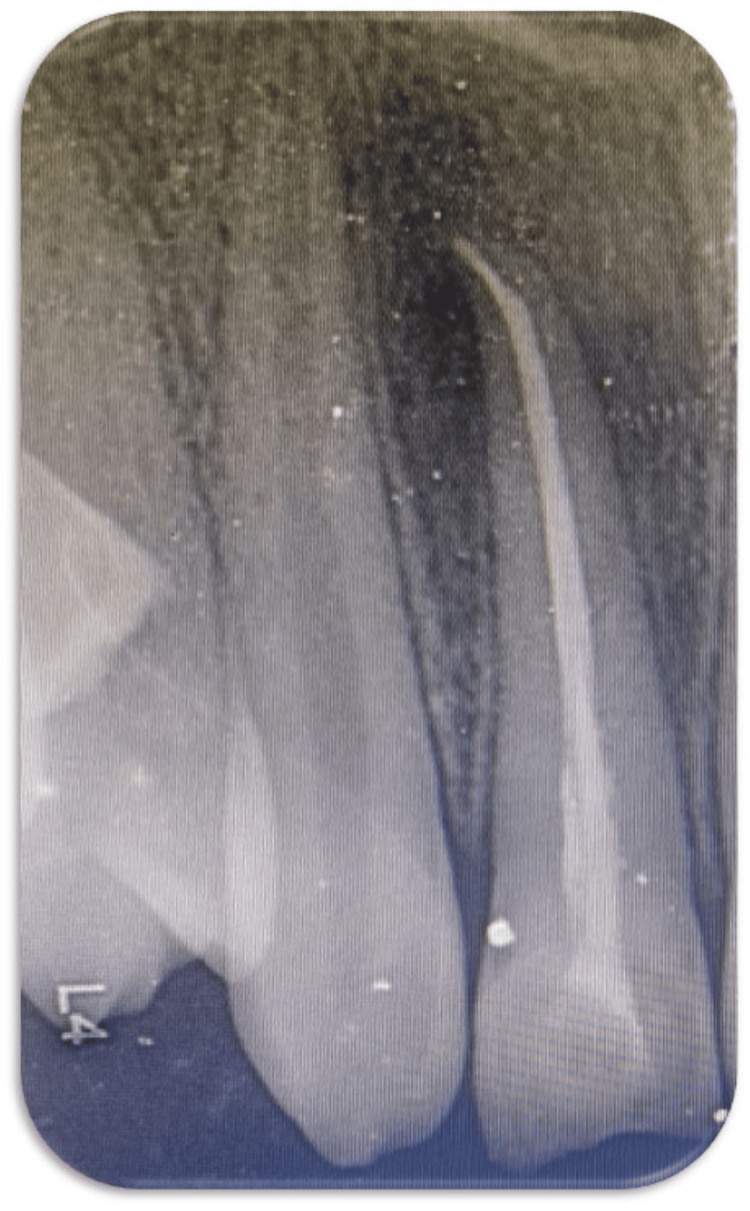
Postoperative radiograph showing root canal treatment with the right permanent maxillary lateral incisor

The treatment planned was endodontic followed by post-endodontic restoration, which was explained to the parents and written consent was taken before starting the procedures. Initially, oral prophylaxis, fluoride application, and pit & fissure sealant application were done during the preventive phase of the treatment. A prophylactic antibiotic regimen was prescribed for three days. At the next appointment, local anesthesia was given and rubber dam isolation was done. Access opening was gained using a BR-31 round burr and an FG 314 safe end burr. No vital was present during the debridement of the canal. Following the measurement of the working length, biomechanical preparation was carried out, and saline and sodium hypochlorite (3%) irrigation was used. Two milliliters of 17% ethylenediaminetetraacetic acid (EDTA), saline, and 2% chlorhexidine were used for the final rinse. The root canal was dried using paper points, and then a calcium hydroxide dressing was applied. After the patient reported that the pain had decreased seven days later, gutta-percha obturation and direct composite restoration were performed. First, the tooth was cleaned, dried, and prepared. Next, the surface was etched for 40 seconds. After that, the tooth was washed, let to air dry, and a bonding agent was applied before it was light-cured for 20 seconds on the prepared surface. The palatal shelf was first built up using a Mylar strip and the rest of the restoration was completed using the composite layering technique from the palatal to the labial side. During the same appointment, direct composite restoration was also done with the right permanent maxillary central incisor. After one week, during the finishing stage, the contouring of the composite resin was performed with the help of various diamond points and burrs. To establish the incisal edge, an ultrafine polishing disc was used, which was followed by final polishing using the polishing points. Post-restorative instructions were given to the child and her parents, and she was recalled for follow-up at one and three months (Figures 2, 3).

## Discussion

The maxillary incisors fracture the most, often in both the primary and permanent dentition. This is mostly seen in teenagers due to their rigorous sports activities [4]. Any non-vital tooth with or without loss of crown structure as a result of trauma is classed as an Ellis class IV fracture, according to the Ellis classification system for teeth fractures. Acute pulpal inflammation and the possibility of microorganisms entering dentinal tubules are both increased by dental injuries [5], making it important to treat the fractured teeth and restore them. In this case, as the tooth was an Ellis class IV fracture and non-vital, the treatment plan was made accordingly. First, the tooth was endodontically treated and calcium hydroxide dressing was given due to its ability to promote the formation of a hard-tissue barrier at the canals apical opening in the coronal fragment and its antibacterial effects, thereby facilitating adequate filling with gutta-percha [6]. Restoration of endodontically treated teeth has always been a concern for dentists, causing an implosion of interest in this field regarding functional and aesthetic problems. For this case, we chose composite restoration for building up the crown after endodontic treatment because of its excellent aesthetics and its ability to conserve tooth structure for a long period of time if maintained properly. The other treatment options may have included the full coverage crown [7]. In the current case, a zirconia crown was not given with the right permanent maxillary lateral incisor as the patient was 11 years old. Before 18 years of age, there is a shift in the gingival margin; therefore, a crown was not advised for the patient, and direct composite restoration was done. The patient's interests and limits, as well as the benefits and drawbacks of each possible technique, should be taken into account while choosing the course of action for treatment.

## Conclusions

A fracture due to trauma in children is one of the most common traumas treated by dentists. Pain, swelling, and aesthetics are some of the most common chief complaints patients report to dental clinics after trauma. After carrying out the examinations and considering the type and severity of the fracture and the emotional and financial status of the patients, we decide on the treatment plan. Usually, if the involvement of the pulp is seen in the traumatized tooth (i.e., Ellis class IV fracture), root canal treatment is done followed by prosthesis placement or permanent restoration. Composite resins have established themselves as one of the most crucial instruments in the physician's toolbox. Achieving a realistic aesthetic result and dependable strength is feasible. The benefit of this technique is directly linked to the professional's dexterity, skill, and technical mastery, as well as the achievement of desirable outcomes.
